# Evolution Dynamics Model of Private Enterprises under Simultaneous and Sequential Innovation Decisions

**DOI:** 10.3390/e25111553

**Published:** 2023-11-17

**Authors:** Chi Zhang, Yutong Wang, Tingqiang Chen

**Affiliations:** School of Economics and Management, Nanjing Tech University, Nanjing 211816, China

**Keywords:** information asymmetry, technology spillover effect, decision-making order, innovation research and development, private enterprises

## Abstract

The innovation of private enterprises plays a crucial role. This study focuses on the impacts of market information asymmetry, the technology spillover effect, and the order of innovation research and development (R&D) decisions on the evolution of private enterprises’ innovation. This study constructs a dynamic model to analyze how the innovation decision-making order of private enterprises influences their profits and intertemporal innovation decision making. First, we derive the equilibrium point under sequential decisions and the stability of the system at the equilibrium point. Second, we investigate the impact of sequential and simultaneous innovation decisions on the evolution of the dynamic system and its economic implications. Finally, we study the evolutionary dynamics of the attractor with the rate of innovation adjustment and point to the existence of multiple equilibria. The results suggest that the speed of the innovation R&D cost change should be moderate, and the asynchronous updating of the innovation R&D strategy can prevent the system evolution from turning into chaos. These conclusions guide innovation policies.

## 1. Introduction

The exogenous growth theory of Solow [1] and the endogenous growth theory represented by Lucas [2], Romer [3], and others have extensively explained that technological innovation is a key driver of economic growth. Innovative research and development (R&D) serves as the engine of technological progress, and it is crucial for the development of private firms. The spillover effects from R&D activities are paramount factors that academics have focused on (Aspremont and Jacquemin [4]; Wang et al. [5]; Hu et al. [6]; Hu et al. [7]; Belitski et al. [8]; Gao et al. [9]). By promoting technological progress, a firm’s R&D activities can make the entire industry highly cost-effective and productive.

Mauleon et al. [10] studied the formation of R&D networks when firms can be either myopic or farsighted, with farsighted firms having additional collaborations on average. The evolution of R&D networks reveals that nearly symmetric networks will be rapidly dismantled, whereas asymmetric networks will persist indefinitely. Qiu R. et al. [11] investigated how innovation depends on the structure of an R&D network and how the structure readjusts based on previous innovation. They constructed an R&D network for firms, each of which calculates its cost and profit in every period and determines the firms that it needs to cooperate with accordingly. However, owing to the path-dependent nature of economic system evolution, R&D networks in many industries have gradually transformed into oligopolistic markets where a few large firms dominate the market share.

In an oligopolistic market, firms producing homogeneous goods must consider the behavioral strategies of other firms when making their production decisions. Additionally, firms consider one another’s innovation R&D statuses and innovation spillovers to determine their optimal innovation R&D strategies. Various improved Cournot models have been developed to analyze the game behavior of oligarchs (Cournot; Rand [12]; Day, [13]; Li et al. [14]; Li et al. [15]; Ding et al. [16]; Mohsin et al. [17]). Li et al. [14] proposed a two-channel game model with different management objectives. Li et al. [15] introduced the Cournot game with conjectural variation and differentiated products. Ding et al. [13] constructed a cooperative duopoly model with implicit collusion. Other studies focused on Gounod’s dynamic model (Agiza [18]; Agliari et al. [19]; Bischi et al. [20]; Bischi and Lamantia [21]), which directly examines the optimal output and system stability with a small number of manufacturers. Askar [22] presented a Cournot duopoly model where competitors aim to maximize their objective functions defined by profits and social welfare. The research explored the stability conditions for the game’s Nash equilibrium and demonstrated that the Nash equilibrium point can become unstable through flip bifurcation.

A completely rational game is typically based on two assumptions: (1) each enterprise has complete information during decision-making, and (2) each enterprise makes decisions based on complete rationality. However, in economic reality, the game among enterprises is continuous, and reaching the Nash equilibrium state immediately is impossible. Additionally, enterprises do not have sufficient information, and corporate decisions are made by individuals who are limited by cognitive abilities and language constraints, resulting in limited rationality (Agiza et al. [18]; Agiza et al. [23]; Long et al. [24]; Williamson [25]). Long et al. [24] examined a dynamic Stackelberg–Cournot duopoly game with one-way spillovers and found that complex dynamic behaviors, such as cycles and chaos, can occur as model parameters vary. Based on this research, we incorporated finite rational production and innovation decisions into the Cournot model and developed a cross-period evolution nonlinear dynamics model to analyze the profitability and survival of firms under finite rational innovation decisions.

In the theoretical calculation part, we incorporated various useful, cutting-edge, and insightful algorithms and mechanisms. Deng et al., Song et al. and Song et al. [26,27,28] proposed optimized parameters for photovoltaic models, utilizing the reverse learning mechanism to generate initial subpopulations, to enhance the convergence velocity, and maintain the population diversity. We would like to express our gratitude to Deng et al. [29] and Deng et al. [30] for their contribution in helping us determine suitable control parameters and select a reasonable mutation strategy for differential evolution to solve real-world engineering optimization problems. Additionally, Grubljesic et al. [31], Namasudra et al. [32], and Shahri et al. [33] provided us with new ideas and insights, which have greatly aided in the construction and analysis of our model.

This study builds upon previous research and focuses on the sequential order of innovation decisions. In the classical game theory, decisions are typically made simultaneously, but this may not be ideal owing to information asymmetry. In line with this, we adopted the approach of Zhou et al. [34] and Zhang et al. [35], where a private enterprise first makes its innovation-decision, specifically determining its R&D investment. Subsequently, another private enterprise can observe the innovation-decision of the first enterprise through certain methods. Hence, the second private enterprise can make an optimal innovation decision based on this information. Our objective is to examine the evolution mechanism of innovation decisions in two private enterprises under this sequential decision-making process and compare it with the traditional simultaneous decision-making scenario. We aim to provide relevant countermeasures and suggestions.

The structure of this paper is organized as follows: Section 2 establishes a two-stage Cournot model with R&D spillover, assuming discrepant products in the market. Section 3 investigates the dynamic model through mathematical calculations, focusing on the stability of equilibrium points and providing stability conditions for these points. Section 4 conducts numerical simulations to analyze bifurcation, stability, routes to chaos, and strange attractors of the system. Finally, Section 5 concludes the study.

## 2. Evolution Model of Innovation Strategy of Two Private Enterprises

We assume that there are two private enterprises in a market, and the index is $i\left(i=1,2\right)$. In addition, the two enterprises produce homogeneous products supplied in the same market. We further assume that horizontal differences exist between the products produced by the two private enterprises to capture the feature of the market competition. Therefore, the inverse demand function can be written as follows:

(1) $$p_{i}(q_{i},q_{j})=a-q_{i}-bq_{j},$$

where $q_{i}$ refers to the selling price set by the enterprise i (which is also applied to j), a refers to the total market share, and b refers to the product differentiation degree of the two enterprises. Specifically, b=0 means that the products of the two enterprises are completely independent and that the output does not affect them; if b=1, the products of the two enterprises can completely replace each other, and if 0<b<1, the products produced by the two enterprises are substitutes for each other, and b describes the degree of substitutability between them.

Innovation is a creative activity that allows enterprises to expand their knowledge base. Within the market, technology enterprises frequently engage in R&D to enhance production efficiency and reduce costs. The competition between private enterprises can typically be described as a two-stage game, which leverages R&D to achieve a cost reduction. In the first stage, all technology enterprises determine the level of R&D investment. Subsequently, in the second stage, these enterprises select the quantity of their products and compete in a Cournot fashion. Owing to the presence of externalities resulting from production and R&D activities, the R&D efforts of private enterprises are often influenced by spillover effects. For instance, the movement of technical personnel or the government’s patent protection policy can facilitate the transfer of technology among private companies. Therefore, the production cost of an enterprise is not only a function of its R&D investment but also of the R&D investment of its competitors:

(2) $$C_{i}(x_{i},x_{j})=c-x_{i}-\beta x_{j},$$

where c is the marginal cost of no R&D effort circumstance, a>c>0, and β measures the innovation spillover effect between firms i and j, which means that the spillover effect will always be symmetrical between two firms and $\beta \in \left[0,1\right]$. The higher the technology spillover coefficient β, the lower the protection degree of enterprise i to the technology patent. β=0 means that the technology of the technology company is confidential; β=1 means that the technology of innovative R&D is completely open to the public. According to Aspremont and Jacquemin [4], the innovation R&D investment of a firm i can be regarded as a quadratic function of the R&D effort, that is, $I(x_{i})=\frac{1}{2}\gamma x_{i}^{2}$. Among them, γ is the cost coefficient of the enterprise i for innovation and R&D. The lower the innovation R&D cost coefficient γ, the stronger the innovation capability of the enterprise i, that is, an enterprise that uses less cost than other enterprises to achieve the same level of innovation and development has a stronger innovation capability. Under the above assumptions, the individual profits of firms 1 and 2 are given as follows:

(3) $$\left\{\begin{array}{l} \pi_{1}(q_{1},q_{2},x_{1},x_{2})=[p_{1}(q_{1},q_{2})-C_{1}(x_{1},x_{2})]q_{1}-\frac{1}{2}\gamma x_{1}^{2}\\ \pi_{2}(q_{1},q_{2},x_{1},x_{2})=[p_{2}(q_{1},q_{2})-C_{2}(x_{1},x_{2})]q_{2}-\frac{1}{2}\gamma x_{2}^{2} \end{array}\right..$$

From Equation (3), the profit of an enterprise is determined by its product output and the level of innovation and R&D efforts. Moreover, the profit of an enterprise is influenced not only by its own output and R&D efforts but also by the product output and innovation R&D efforts of its competitors. By substituting Equations (1) and (2) into Equation (3), we can derive the following outcomes:

(4) $$\left\{\begin{array}{l} \pi_{1}(q_{1},q_{2},x_{1},x_{2})=[(a-q_{1}-bq_{2})-(c-x_{1}-\beta x_{2})]q_{1}-\frac{1}{2}\gamma x_{1}^{2}\\ \pi_{2}(q_{1},q_{2},x_{1},x_{2})=[(a-q_{2}-bq_{1})-(c-x_{2}-\beta x_{1})]q_{2}-\frac{1}{2}\gamma x_{2}^{2} \end{array}\right..$$

To find the optimal output of the first stage game, the first derivative of the profit function of the enterprise concerning its product output is obtained:

(5) $$\left\{\begin{array}{l} \frac{\partial \pi_{1}}{\partial q_{1}}=a-2q_{1}-bq_{2}-c+x_{1}+\beta x_{2}\\ \frac{\partial \pi_{2}}{\partial q_{2}}=a-2q_{2}-bq_{1}-c+x_{2}+\beta x_{1} \end{array}\right..$$

As shown in Equation (5), to solve the optimal production level, let $\partial \pi_{i}/\partial q_{i}=0$ and i=1,2. Through calculation, we can obtain the perfect Nash equilibrium of the second-stage sub-game:

(6) $$\left\{\begin{array}{l} q_{1}^{*}=\frac{(a-c)(2-b)+(2-b\beta )x_{1}+(2\beta -b)x_{2}}{4-b^{2}}\\ q_{2}^{*}=\frac{(a-c)(2-b)+(2\beta -b)x_{1}+(2-b\beta )x_{2}}{4-b^{2}} \end{array}\right.$$

Then, the profit functions of enterprises 1 and 2 can be rewritten as the functions of their R&D efforts by substituting the equilibrium output of Equation (6) into Equation (4):

(7) $$\left\{\begin{array}{l} \pi_{1}(x_{1},x_{2})=\frac{{\left[(a-c)(2-b)+(2-b\beta )x_{1}+(2\beta -b)x_{2}\right]}^{2}}{{\left(4-b^{2}\right)}^{2}}-\frac{1}{2}\gamma x_{1}^{2}\\ \pi_{2}(x_{1},x_{2})=\frac{{\left[(a-c)(2-b)+(2\beta -b)x_{1}+(2-b\beta )x_{2}\right]}^{2}}{{\left(4-b^{2}\right)}^{2}}-\frac{1}{2}\gamma x_{2}^{2} \end{array}\right..$$

By differentiating Equation (7) with respect to enterprises 1 and 2, respectively, we can obtain a local estimate of the marginal profit of the R&D efforts:

(8) $$\left\{\begin{array}{l} \frac{\partial \pi_{1}}{\partial x_{1}}=\frac{2(2-b\beta )[(a-c)(2-b)+(2-b\beta )x_{1}+(2\beta -b)x_{2}]}{{\left(4-b^{2}\right)}^{2}}-\gamma x_{1}\\ \frac{\partial \pi_{2}}{\partial x_{2}}=\frac{2(2-b\beta )[(a-c)(2-b)+(2\beta -b)x_{1}+(2-b\beta )x_{2}]}{{\left(4-b^{2}\right)}^{2}}-\gamma x_{2} \end{array}\right..$$

Owing to the lack of complete market information, enterprises are unable to engage in completely rational market competition. They often lack access to crucial data, such as the actual market demand, output, and R&D efforts of their competitors. Consequently, these companies tend to exhibit a certain degree of short-sightedness. To maximize their profits, these short-sighted private enterprises make adjustments to their investments in innovation and R&D. We assume that, in period t+1, if the marginal profit of enterprise i in phase t+1 is positive, that is, $\partial \pi_{i}/\partial x_{i}>0$, then enterprise i increases its R&D investment in period t+1. On the contrary, if $\partial \pi_{i}/\partial x_{i}<0$ in phase t, firm i will reduce its R&D investment in phase t+1. To greatly understand the influence of information asymmetry on innovation and research in private enterprises, we conducted a study where enterprise 1 made its innovation decision first. Subsequently, enterprise 2 acquired information about enterprise 1’s decision, including the amount of their innovation and research investments. Based on this information, enterprise 2 then made its own innovation decision.

(9) $$\left\{\begin{array}{l} x_{1}(t+1)=x_{1}(t)+\alpha_{1}x_{1}(t)\frac{\partial \pi_{1}}{\partial x_{1}}(x_{1}(t),x_{2}(t))\\ x_{2}(t+1)=x_{2}(t)+\alpha_{2}x_{2}(t)\frac{\partial \pi_{2}}{\partial x_{2}}(x_{1}(t+1),x_{2}(t)) \end{array}\right..$$

By substituting Equation (8) into Equation (9), we can obtain the dynamic model of innovation R&D investment of two enterprises as follows:

(10) $$\left\{\begin{array}{l} x_{1}(t+1)=x_{1}(t)+\alpha_{1}x_{1}(t)\{\frac{2(2-b\beta )[(a-c)(2-b)+(2-b\beta )x_{1}(t)+(2\beta -b)x_{2}(t)]}{{\left(4-b^{2}\right)}^{2}}-\gamma x_{1}(t)\}\\ x_{2}(t+1)=x_{2}(t)+\alpha_{2}x_{2}(t)\{\frac{2(2-b\beta )[(a-c)(2-b)+(2\beta -b)x_{1}(t+1)+(2-b\beta )x_{2}(t)]}{{\left(4-b^{2}\right)}^{2}}-\gamma x_{2}(t)\} \end{array}\right..$$

In this formula, $\alpha_{i}$ is the adjustment speed of enterprise i. The enterprise readjusts its R&D efforts according to its marginal profit in period t+1 during period t. Suppose $\alpha_{i}>0$, and assume a>0 represents the market size; b represents the degree of product differentiation, where $b\in \left[0,1\right]$. c represents the marginal costs without R&D efforts, where a>c>0. β measures the spillover effect between firms i and j, $\beta \in \left[0,1\right]$. To simplify the expression, we make R=2(2−b)(a−c)(2−bβ), T=2(2β−b)(2−bβ), $S={\left(4-b^{2}\right)}^{2}$, and $U=2{\left(2-b\beta \right)}^{2}-\gamma {\left(4-b^{2}\right)}^{2}$.

Then, Equation (10) can be simplified to the following form:

(11) $$\left\{\begin{array}{l} x_{1}(t+1)=x_{1}\left(t\right)+\alpha_{1}x_{1}\left(t\right)(\frac{U}{S}x_{1}\left(t\right)+\frac{T}{S}x_{2}\left(t\right)+\frac{R}{S})\\ x_{2}(t+1)=x_{2}\left(t\right)[\frac{\alpha_{1}\alpha_{2}TU}{S^{2}}x_{1}^{2}\left(t\right)+\frac{\alpha_{1}\alpha_{2}T^{2}}{S^{2}}x_{1}\left(t\right)x_{2}\left(t\right)+(\alpha_{2}+\frac{\alpha_{1}\alpha_{2}R}{S})x_{1}\left(t\right)+\frac{\alpha_{2}U}{S}x_{2}\left(t\right)+1+\frac{\alpha_{2}R}{S}] \end{array}\right..$$

## 3. Stability Analysis of the Model

### 3.1. Existence of Fixed Points

The two-dimensional map for the system in Equation (11) takes the following form:

(12) $$\left\{\begin{array}{l} x_{1}\mapsto x_{1}+\alpha_{1}x_{1}(\frac{U}{S}x_{1}+\frac{T}{S}x_{2}+\frac{R}{S})\\ x_{2}\mapsto x_{2}[\frac{\alpha_{1}\alpha_{2}TU}{S^{2}}x_{1}^{2}+\frac{\alpha_{1}\alpha_{2}T^{2}}{S^{2}}x_{1}x_{2}+(\alpha_{2}+\frac{\alpha_{1}\alpha_{2}R}{S})x_{1}+\frac{\alpha_{2}U}{S}x_{2}+1+\frac{\alpha_{2}R}{S}] \end{array}\right..$$

To greatly understand the intricate dynamic behavior of the system in Equation (11), first, the equilibrium point of the model should be examined, and its stability in the vicinity of this point must be assessed. The fixed points of the map in Equation (12) can be determined by solving the following equations:

(13) $$\left\{\begin{array}{l} x_{1}=x_{1}+\alpha_{1}x_{1}(\frac{U}{S}x_{1}+\frac{T}{S}x_{2}+\frac{R}{S})\\ x_{2}=x_{2}[\frac{\alpha_{1}\alpha_{2}TU}{S^{2}}x_{1}^{2}+\frac{\alpha_{1}\alpha_{2}T^{2}}{S^{2}}x_{1}x_{2}+(\alpha_{2}+\frac{\alpha_{1}\alpha_{2}R}{S})x_{1}+\frac{\alpha_{2}U}{S}x_{2}+1+\frac{\alpha_{2}R}{S}] \end{array}\right..$$

By applying a simple calculation, we could obtain the four equilibrium points, $E_{1}(0,0)$, $E_{2}(0,-\frac{R}{U})$, $E_{3}(-\frac{R}{U},0)$, and $E_{4}(-\frac{R}{U+T},-\frac{R}{U+T})$, by solving the equation.

### 3.2. Stability of Fixed Points and Bifurcations

The equilibrium points $E_{1}$, $E_{2}$, and $E_{3}$ are the boundary equilibrium points, where $E_{4}$ is the only Nash equilibrium point. To focus on the economic significance, the four equilibrium points should be non-negative. From the previous analysis, the parameters satisfy a>c, 0≤b≤1, 0≤β≤1, and R>0. By solving the inequality, all equilibrium points are non-negative when the parameters U and T satisfy the following condition:

(14) $$\left\{\begin{array}{l} U<0\\ U+T<0 \end{array}\right..$$

To study the local stability of the equilibrium point, we calculate the Jacobian matrix of the map evaluated at point $\left(x_{1},x_{2}\right)$, which is given by the following equation:

(15) $$J=\left[\begin{array}{ll} 1+\alpha_{1}(\frac{R}{S}+\frac{2U}{S}x_{1}+\frac{T}{S}x_{2}) & \alpha_{1}x_{1}\frac{T}{S}\\ \alpha_{2}x_{2}[\frac{2\alpha_{1}TU}{S^{2}}x_{1}+\frac{\alpha_{1}T^{2}}{S^{2}}x_{2}+(1+\frac{\alpha_{1}R}{S})] & \frac{\alpha_{1}\alpha_{2}TU}{S^{2}}x_{1}^{2}+\frac{2\alpha_{1}\alpha_{2}T^{2}}{S^{2}}x_{1}x_{2}+(\alpha_{2}+\frac{\alpha_{1}\alpha_{2}R}{S})x_{1}+\frac{2\alpha_{2}U}{S}x_{2}+(1+\frac{\alpha_{2}R}{S}) \end{array}\right].$$

To determine the stability of the equilibrium point, we calculate the eigenvalue of the Jacobian matrix at these points below.

**Proposition** **1.** *The boundary equilibrium point* $E_{1}$ *is a repelling node point.*

**Proof.**  For the boundary equilibrium point $E_{1}(0,0)$, set $x_{1}=0$ and $x_{2}=0$; then, the Jacobian matrix in Equation (12) becomes

(16) $$J(E_{1})=\left[\begin{array}{ll} 1+\alpha_{1}\frac{R}{S} & 0\\ 0 & 1+\alpha_{2}\frac{R}{S} \end{array}\right].$$

Considering that R>0 and S>0, the absolute values of the eigenvalues of the Jacobian matrix at equilibrium point $E_{1}$ are $\left|\lambda_{1}\right|=(1+\alpha_{1}\frac{R}{S})>1$ and $\left|\lambda_{2}\right|=(1+\alpha_{2}\frac{R}{S})>1$, so the equilibrium point $E_{1}=\left(0,0\right)$ is the repelling node point. □

**Proposition** **2.** *For boundary equilibrium point* $E_{2}(0,-\frac{R}{U})$*:*

*(i)*  *As* U−T<0*, the equilibrium point* $E_{2}$ *is unstable.*
*(ii)*  *As* U−T>0*,* $0<\alpha_{1}<\frac{-2SU}{R\left(U-T\right)}$

*If* $\alpha_{2}>\frac{2S}{R}$*, equilibrium point* $E_{2}$ *is an unstable saddle point.*

*If* $\alpha_{2}<\frac{2S}{R}$*, equilibrium point* $E_{2}$ *is a stable node point.*

*(iii)*  *As* U−T>0*,* $\alpha_{1}>\frac{-2SU}{R\left(U-T\right)}$*, and equilibrium point* $E_{2}$ *is an unstable point.*

**Proof.**  For the boundary equilibrium point $E_{2}$, the Jacobian matrix can be written as follows:

(17) $$J(E_{2})=\left[\begin{array}{ll} 1+\alpha_{1}\frac{R(U-T)}{US} & 0\\ \frac{-\alpha_{2}RT}{US} & 1-\frac{\alpha_{2}R}{S} \end{array}\right].$$

The eigenvalues of the Jacobian matrix at the boundary equilibrium point $E_{2}$ are $\lambda_{1}=1+\alpha_{1}\frac{R(U-T)}{SU}$ and $\lambda_{2}=1-\frac{\alpha_{2}R}{S}$.

If U−T<0, the absolute value of the eigenvalue is $\left|\lambda_{1}\right|=\left|1+\alpha_{1}\frac{R(U-T)}{SU}\right|>1$. Therefore, when U−T<0, equilibrium point $E_{2}$ is unstable.

Otherwise, if U−T>0 and $0<\alpha_{1}<\frac{-2SU}{R\left(U-T\right)}$, the absolute value of the eigenvalue is $\left|\lambda_{1}\right|=\left|1+\alpha_{1}\frac{R\left(U-T\right)}{SU}\right|<1$. In this case, if $\alpha_{2}>\frac{2S}{R}$, the absolute value of the eigenvalue is $\left|\lambda_{2}\right|=\left|1-\frac{\alpha_{2}R}{S}\right|>1$, so the equilibrium point $E_{2}$ is an unstable saddle point; if $\alpha_{2}<\frac{2S}{R}$, the absolute value of the eigenvalue is $\left|\lambda_{2}\right|=\left|1-\frac{\alpha_{2}R}{S}\right|<1$, and the equilibrium point $E_{2}$ is a stable node point.

Finally, if U−T>0 and $\alpha_{1}>\frac{-2SU}{R(U-T)}$, the absolute value of the eigenvalue is $\left|\lambda_{1}\right|=\left|1+\alpha_{1}\frac{R\left(U-T\right)}{SU}\right|>1$; hence, equilibrium point $E_{2}$ is unstable.

For boundary equilibrium point $E_{3}$, the case is almost the same as equilibrium point $E_{2}$. The Jacobian matrix is the upper triangular matrix, which can be expressed as follows:

(18) $$J(E_{3})=\left[\begin{array}{ll} 1-\frac{\alpha_{1}R}{S} & \frac{-\alpha_{1}RT}{US}\\ 0 & 1+\alpha_{2}\frac{R(U-T)}{SU} \end{array}\right].$$

The two real eigenvalues of the Jacobian matrix at boundary equilibrium point $E_{3}$ are $\lambda_{1}=1-\frac{\alpha_{1}R}{S}$ and $\lambda_{2}=1+\alpha_{2}\frac{R(U-T)}{SU}$. The discussion for $E_{2}$ also applies to $E_{3}$ because their coordinates are symmetric. □

From a mathematical perspective, the local stability of equilibrium points $E_{1}$, $E_{2}$, and $E_{3}$ should be analyzed, including their behaviors at the boundary of the local stable region. However, notably, these equilibriums correspond to scenarios where one or both firms exit the market, resulting in a transition from a duopoly to a monopoly market. Our main focus is to examine the evolution of a duopoly market as the parameters change. Therefore, we will solely concentrate on analyzing the local stability, bifurcation, and chaos of the Nash equilibrium $E_{4}$.

By setting $x_{1}=x_{2}=-\frac{R}{U+T}$, we obtain the Jacobian matrix at point $E_{4}(-\frac{R}{U+T},-\frac{R}{U+T})$:

(19) $$J(E_{4})=\left[\begin{array}{ll} 1+\alpha_{1}\frac{R+2U+T}{S}\frac{-R}{U+T} & \alpha_{1}\frac{-R}{U+T}\frac{T}{S}\\ \frac{2\alpha_{1}\alpha_{2}TU+\alpha_{1}\alpha_{2}T^{2}}{S^{2}}{\frac{-R}{U+T}}^{2}+(\alpha_{2}+\frac{\alpha_{1}\alpha_{2}R}{S})\frac{-R}{U+T} & \frac{\alpha_{1}\alpha_{2}TU+2\alpha_{1}\alpha_{2}T^{2}}{S^{2}}{\frac{-R}{U+T}}^{2}+\frac{\alpha_{2}S+\alpha_{1}\alpha_{2}R+2\alpha_{2}U}{S}\frac{-R}{U+T}+(1+\frac{\alpha_{2}R}{S}) \end{array}\right].$$

The theoretical calculation would be so complex that we directly go to the simulation part to show the dynamic features of $E_{4}$.

## 4. Numerical Simulation Results

Numerical simulation methods form the foundation of a nonlinear dynamic analysis. In this section, we will perform various numerical calculations to support our analysis. Specifically, we will present the bifurcation diagram along with the corresponding largest Lyapunov exponent, including the evolution of attractors and basins of attraction. These diagrams will allow us to observe numerous typical features.

### 4.1. Bifurcation Diagram

In the previous section, we focused on examining the local stability and local bifurcation of the equilibrium points. In this section, we will shift our focus to analyzing the dynamic behavior of the model using the numerical simulation method.

First, we would fix the parameters to a=61, b=0.85, c=51.5, β=0.75, and γ=0.8, and then investigate the influences of $\alpha_{1}$ and $\alpha_{2}$ (the speeds of adjustment for two firms) on the evolution dynamics of innovation R&D investment under sequential and simultaneous decision situations. Specifically, we would fix the value $\alpha_{1}$ at 0.45, divide $\alpha_{2}$ into 1000 parts in an interval, $\left[0,0.5\right]$ or $\left[0,0.65\right]$ (depending on whether the value of $x_{1}$ goes infinite), and draw the bifurcation diagram of evolution dynamics concerning parameter $\alpha_{2}$ under sequential decision and simultaneous decision, respectively.

**Figure 1:**
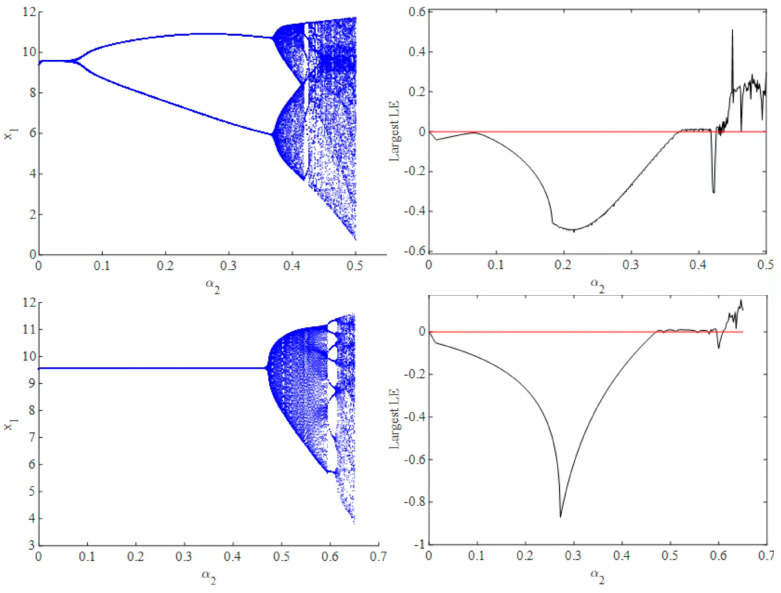
Bifurcation diagram with respect to $\alpha_{2}$ under simultaneous decision and sequential decision ($\alpha_{1}=0.45$).

Figure 1 shows that in the case of simultaneous versus sequential decision making, the bifurcation diagram shows a completely different pattern. In the case of simultaneous decision making, as shown in Figure 1, when we fix $\alpha_{1}=0.45$ and draw the bifurcation diagram, a flip bifurcation is displayed when $\alpha_{2}$ is approximately 0.1. Before $\alpha_{2}$ increases to approximately 0.35, the dynamic system in Equation (11) is in a stable period-2 state. Then, when $\alpha_{2}$ is bigger than approximately 0.35, the system turns into a quasi-periodic state, so there is a Neimark–Sacker bifurcation. Then, the system turns into a chaotic state. Figure 1 shows the largest Lyapunov exponent with varied $\alpha_{2}$. When the largest Lyapunov exponent is less than zero, the system is in a stable state, whereas when it equals zero, bifurcation occurs. Finally, when the largest Lyapunov exponent is larger than zero, the system turns into chaos.

With regard to sequential decision making, as shown in Figure 1, the whole system can stay at Nash equilibrium, whereas $\alpha_{2}$ is less than approximately 0.47. With the increase in $\alpha_{2}$, the system then goes into a chaotic state. In addition, Figure 1 shows that the largest Lyapunov exponent is less than zero, so the system goes back to the stable state when $\alpha_{2}$ is approximately 5.95. However, there are seven different stable nodes, which are called multi-stability and will be analyzed later.

After completing the mathematical description of the nonlinear dynamic system, we delve into its economic implications. First, Figure 1 shows that a low adjustment speed can contribute to system stabilization. As the adjustment speed increases, the system tends to exhibit chaotic behavior, implying that the market dynamics become highly unpredictable. Consequently, market participants may find it challenging to make informed decisions regarding purchase volumes, production volumes, and prices, resulting in reduced market efficiency. Second, when comparing the simulation results of simultaneous and sequential decision making, we observe that the system can maintain stability at Nash equilibrium for large values of $\alpha_{2}$ when sequential decision making is implemented.

### 4.2. Evolution of Attractors

Studying nonlinear systems is crucial to understand their final states. Attractors play a significant role in revealing the asymptotic behavior of a nonlinear dynamic system as the number of iterations approaches infinity. To carry this out, we still fix the parameters as a=61, b=0.85, c=51.5, β=0.75, γ=0.8, and $\alpha_{1}=0.45$ and study the effect of the adjustment speed $\alpha_{2}$ on the dynamic system under sequential innovative decision.

**Figure 2:**
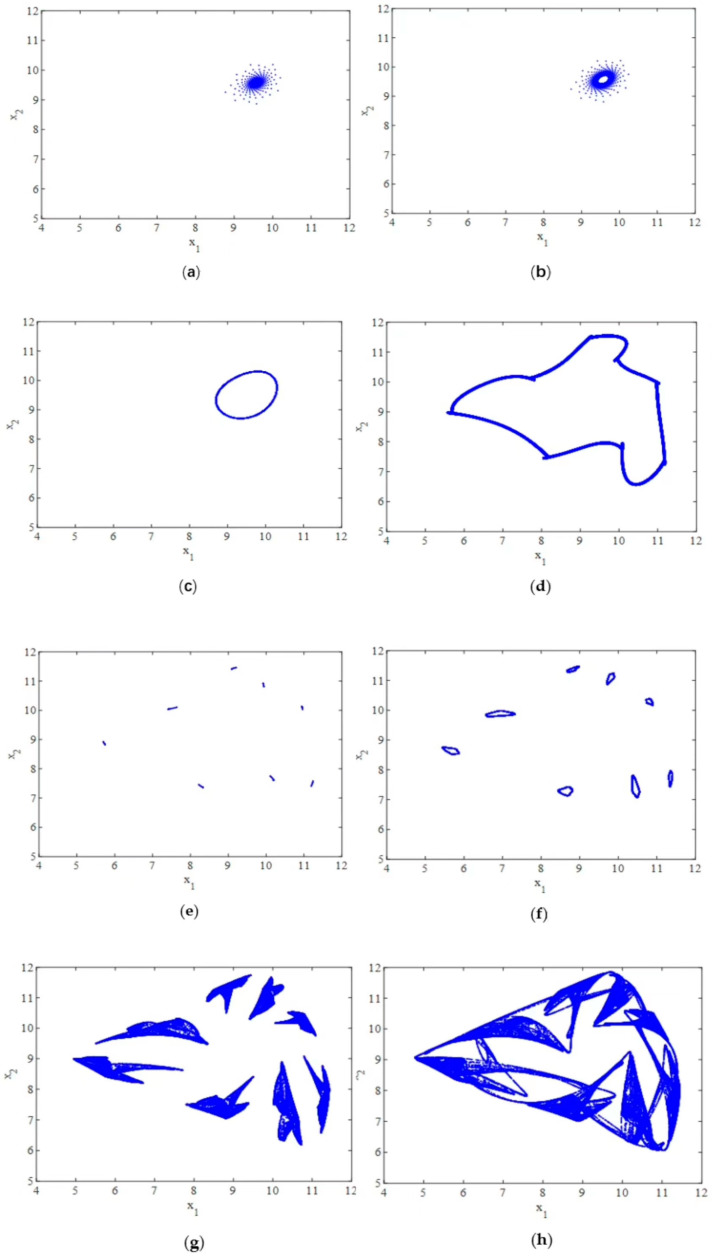
(**a**) The attractor diagram when $\alpha_{2}=0.468$. (**b**) The attractor diagram when $\alpha_{2}=0.4703$. (**c**) The attractor diagram when $\alpha_{2}=0.48$. (**d**) The attractor diagram when $\alpha_{2}=0.59623$. (**e**) The attractor diagram when $\alpha_{2}=0.5965$. (**f**) The attractor diagram when $\alpha_{2}=0.613$. (**g**) The attractor diagram when $\alpha_{2}=0.616$. (**h**) The attractor diagram when $\alpha_{2}=0.618$.

Figure 2a shows that when parameter $\alpha_{2}=0.468$, the attractor is a period-1 focus with rough selvage. With the increase in $\alpha_{2}$, the period-1 focus turns into an invariant cycle with rough selvage through Neimark–Sacker bifurcation, as shown in Figure 2b. Then, the invariant cycle grows in size, and the rough selvage vanishes (Figure 2c). The shape of the invariant cycle continues to change with an increase in $\alpha_{2}$, as shown in Figure 2d. As shown in Figure 2e, when $\alpha_{2}$ equals 0.5965, the invariant cycle breaks and forms an eight-piece attractor.

After that, each piece individually forms an invariant cycle at $\alpha_{2}=0.613$ (Figure 2f). Then, the attractor turns into chaos (Figure 2g) and finally forms a connected chaotic attractor (Figure 2h).

The coexistence of multiple equilibria in economies is an intriguing and significant phenomenon. The stability of an equilibrium point is influenced by the initial conditions, leading to path dependence in economics. This path dependence can result in multiple bifurcations at the same level, giving rise to complex dynamic behaviors, such as the coexistence of multiple attractors, fractals, and chaos. The presence of multiple attractors indicates the existence of multiple steady states in the system. In other words, the bifurcation of a nonlinear system can cause the number of solutions to change with variations in the parameters and initial conditions. Therefore, the study of multi-steady motion is closely linked to a bifurcation analysis. By examining the coexistence of multiple attractors and their corresponding basins of attraction, we can gain insights into the global dynamic behavior of the system in Equation (11).

Furthermore, the simulation results highlight the high sensitivity of the system’s solution to the initial values. Accurately identifying the current state of the system can be immensely valuable in managing and facilitating the evolution of innovation. This understanding can aid in formulating rational policies that enable firms to operate optimally by utilizing efficient inputs and outputs.

## 5. Conclusions

This study presents a two-stage dynamic game model that considers asymmetric information and innovation R&D spillovers between two private enterprises. This study focuses on analyzing how one player’s knowledge of the other’s behavior affects the stability of the Cournot–Nash equilibrium in the market.

In the first stage, both enterprises determine their R&D efforts to lower production costs. In the second stage, they compete in a Cournot competition market and decide their output levels. To account for the impact of sequential innovation decisions resulting from information asymmetry, the model allows enterprise 1 to make its innovation decision first, with enterprise 2 subsequently learning about enterprise 1’s decision through certain means. Enterprise 2 then makes its own innovation decision based on this information to maximize its profits.

A theoretical analysis demonstrates that information serves to stabilize the Nash equilibrium and suppress chaotic behavior. Bifurcation diagrams, the largest Lyapunov exponent, and the evolution of attractors are employed to examine the dynamics of the model. The findings indicate that acquiring information about the strategies of other enterprises can enhance the stability of the Nash equilibrium in the market of the two private enterprises. Numerical simulations further demonstrate that updating strategies asynchronously can prevent chaotic behavior. These results have important implications for government policymakers seeking to develop innovation policies that stabilize the market and promote social innovation.

## Data Availability

Data are contained within the article.
